# Prediction of progression from mild cognitive impairment to Alzheimer's disease with longitudinal and multimodal data

**DOI:** 10.3389/frdem.2023.1271680

**Published:** 2023-11-24

**Authors:** Huitong Ding, Biqi Wang, Alexander P. Hamel, Mark Melkonyan, Ting F. A. Ang, Rhoda Au, Honghuang Lin

**Affiliations:** ^1^Department of Anatomy and Neurobiology, Boston University Chobanian & Avedisian School of Medicine, Boston, MA, United States; ^2^The Framingham Heart Study, Boston University Chobanian & Avedisian School of Medicine, Boston, MA, United States; ^3^Department of Medicine, University of Massachusetts Chan Medical School, Worcester, MA, United States; ^4^Slone Epidemiology Center, Boston University Chobanian & Avedisian School of Medicine, Boston, MA, United States; ^5^Department of Epidemiology, Boston University School of Public Health, Boston, MA, United States; ^6^Departments of Neurology and Medicine, Boston University Chobanian & Avedisian School of Medicine, Boston, MA, United States

**Keywords:** MCI-to-AD progression, longitudinal data, multimodal data, machine learning, prediction

## Abstract

**Introduction:**

Accurate prediction of the progression from mild cognitive impairment (MCI) to Alzheimer's disease (AD) within a certain time frame is crucial for appropriate therapeutic interventions. However, it is challenging to capture the dynamic changes in cognitive and functional abilities over time, resulting in limited predictive performance. Our study aimed to investigate whether incorporating longitudinal multimodal data with advanced analytical methods could improve the capability to predict the risk of progressing to AD.

**Methods:**

This study included participants from the Alzheimer's Disease Neuroimaging Initiative (ADNI), a large-scale multi-center longitudinal study. Three data modalities, including demographic variables, neuropsychological tests, and neuroimaging measures were considered. A Long Short-Term Memory (LSTM) model using data collected at five-time points (baseline, 6, 12, 18, and 24-month) was developed to predict the risk of progression from MCI to AD within 2 years from the index exam (the exam at 24-month). In contrast, a random forest model was developed to predict the risk of progression just based on the data collected at the index exam.

**Results:**

The study included 347 participants with MCI at 24-month (age: mean 75, SD 7 years; 39.8% women) from ADNI, of whom 77 converted to AD over a 2-year follow-up period. The longitudinal LSTM model showed superior prediction performance of MCI-to-AD progression (AUC 0.93 ± 0.06) compared to the random forest model (AUC 0.90 ± 0.09). A similar pattern was also observed across different age groups.

**Discussion:**

Our study suggests that the incorporation of longitudinal data can provide better predictive performance for 2-year MCI-to-AD progression risk than relying solely on cross-sectional data. Therefore, repeated or multiple times routine health surveillance of MCI patients are essential in the early detection and intervention of AD.

## 1 Introduction

Mild cognitive impairment (MCI) is a syndrome characterized by cognitive decline, but not to the extent that it significantly affects a person's daily activities (Werner and Korczyn, 2008). MCI is considered an intermediate and dynamic stage between normal aging and dementia (Mielke et al., 2014). Individuals diagnosed with MCI are at an increased risk of developing Alzheimer's disease (AD) (Albert et al., 2011), with a progression rate of 10–15% per year (Petersen et al., 1999). The American Academy of Neurology acknowledges the significance of individuals with MCI as a clinical population that needs to be identified and monitored (Petersen et al., 2001). However, MCI is a very heterogeneous disease and many patients might not progress for many years (Jack Jr et al., 2013). Therefore, identifying MCI patients, who are at risk of rapid progression to AD, is a crucial step in initiating timely interventions to prevent or slow down the AD onset.

Emerging research has utilized a variety of cross-sectional data to predict the progression from MCI to AD (Ye et al., 2012; Wee et al., 2013; Li et al., 2016). However, these models may be insufficient to capture the dynamic changes in cognitive and functional abilities that occur over time in individuals with MCI, which can limit their accuracy in predicting MCI to AD progression. Neuropsychological (NP) tests and brain magnetic resonance imaging (MRI) measures are two primary clinical indices of neurodegeneration. Studies have shown that these indicators exhibit longitudinal changes that are helpful in diagnosing dementia (Fox and Schott, 2004; Nation et al., 2019). Therefore, it would be interesting to harness data collected at multiple time points to predict the risk of progression from MCI to AD.

Longitudinal clinical exams can be challenging to analyze due to the heterogeneity of AD progression. Traditional methods of risk prediction, such as random forest models, have limited capacity to capture complex temporal patterns and dependencies across multiple time points. These methods usually rely on aggregating the data across different time points, which may result in a loss of important information reflecting the fluidity of disease expression. Prior research utilizing longitudinal data to predict MCI to AD progression has shown constrained predictive efficacy (Misra et al., 2009; Er and Goularas, 2020). Recent advances in deep learning networks, such as the Long Short-Term Memory (LSTM) models, provide a promising solution to better capture the temporal dynamics of longitudinal data. The ease of data acquisition also plays a pivotal role in developing longitudinal models. For instance, positron emission tomography (PET) data collection incurs relatively high costs and poses challenges in obtaining longitudinal data. Consequently, models relying on PET data may not be directly applicable in more general populations (Zhang et al., 2012).

Using longitudinal data collected from the Alzheimer's Disease Neuroimaging Initiative (ADNI), the objectives of this study were to determine whether: (a) incorporating data at multiple time points could improve the predictive performance of MCI to AD progression than single time point prediction; (b) clinical manifestations (NP tests), neuroimaging biomarkers (MRI measures), and well-documented demographic risk factors (age, sex, education) capture complementary information and can improve the prediction of MCI-to-AD progression.

## 2 Materials and methods

### 2.1 Study population

Data used in the preparation of this article were obtained from the Alzheimer's Disease Neuroimaging Initiative (ADNI) database (adni.loni.usc.edu). The ADNI was launched in 2003 as a public-private partnership, led by Principal Investigator Michael W. Weiner, MD (Carrillo et al., 2012). The primary goal of ADNI has been to test whether serial MRI, positron emission tomography, other biological markers, and clinical and neuropsychological assessment can be combined to measure the progression of MCI and early AD.

The ADNI Participants had repeated clinical examinations, including a neuropsychological assessment and MRI scan, approximately every 6 months. The study utilized several measures including subjective memory concern, the Logical Memory Test (LMT), Mini-Mental State Exam (MMSE), Clinical Dementia Rating scale (CDR), and the National Institute of Neurological and Communicative Disorders and Stroke and the Alzheimer's Disease and Related Disorders Association Alzheimer's Criteria (NINCDS-ADRDA) to diagnose MCI and AD. More details on ADNI's diagnostic criteria and methods can be found in General Procedures Manual on the ADNI website^1^. For participants with missing diagnosis at a specific exam, we interpolated the diagnosis by the status from the immediate prior and follow up exams if the diagnosis was consistent. For example, if the diagnoses at the immediate prior exam and the follow up exam were both MCI, the diagnosis for the current exam was assumed as MCI.

**Figure 1:**
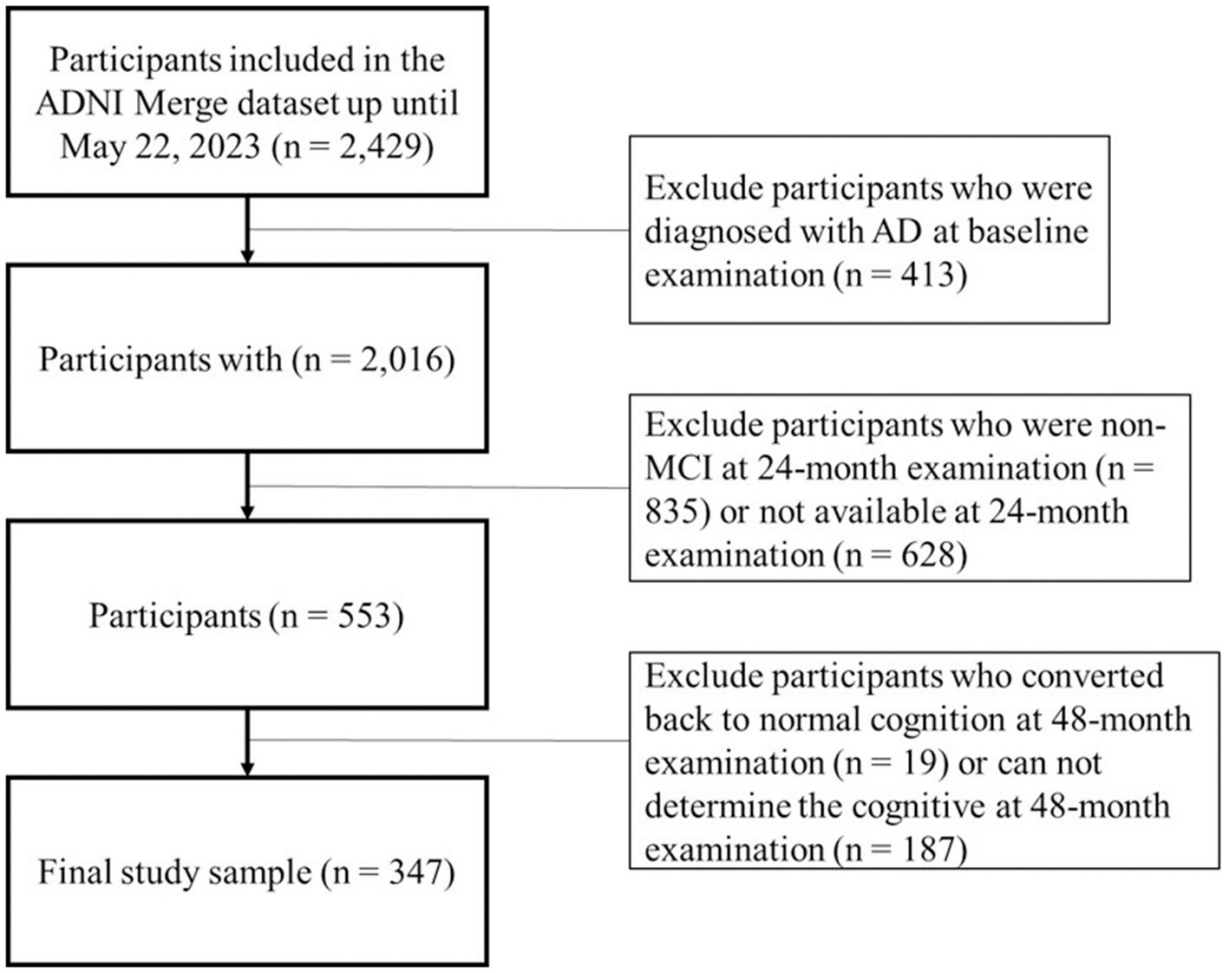
Sample selection diagram of this study.

This study utilized the ADNI-Merge dataset (IDA, 2023), which was downloaded in May 2023 for the current analysis. The ADNI Merge dataset combines data from the ADNI-1, ADNI-GO, and ADNI-2 studies, accumulating to over 2,400 participants. We included only participants who were non-AD (cognitively intact or MCI) at the baseline examination (*n* = 2,016). We then restricted to participants who had corresponding NP tests and MRI measures collected at five time points (baseline, 6, 12, 18, and 24-month) and were diagnosed with MCI at 24-month (*n* = 553). The index exam is defined as the exam at 24-month, and the outcome is defined as the progression from MCI to AD before 48-month. The participants who either reverted to normal cognitive status (*n* = 19) or cannot determine the cognitive status at the 48-month follow-up examination (*n* = 187) were excluded. The remaining participants who converted to AD (MCI converts, MCI-C for short) or remain MCI (MCI non-converters, MCI-NC for short) at 48-month were included as the final samples (*n* = 347) (Figure 1). The study design adhered to the TRIPOD (Transparent Reporting of a multivariable prediction model for Individual Prognosis Or Diagnosis) statement (Collins et al., 2015).

### 2.2 Neuropsychological assessment

Details of the neuropsychological assessment in ADNI have been previously described (Aisen et al., 2010). In this study, we included 15 NP tests such as the functional assessment questionnaire (FAQ) (Pfeffer et al., 1982); the sum of boxes of the clinical dementia rating (CDR-SB) (Morris, 1993); Alzheimer's Disease Assessment Scale-Cognitive Subscale 11 (ADAS-cog 11), Subscale 13 (ADAS-cog 13), and Delayed Word Recall (ADAS-Q4) (Rosen et al., 1984); Mini-Mental State Examination (MMSE) (Folstein et al., 1975); Montreal Cognitive Assessment (MoCA) (Nasreddine et al., 2005); logical memory delayed recall total (LDELTOTAL) score (Wechsler, 1987); the Modified Preclinical Alzheimer Cognitive Composite with Trail Making Test B (mPACC-trailsB) and Digit Symbol Substitution (mPACC-digit) (Donohue et al., 2014); Trail Making Test B (Trails B) (Tombaugh, 2004); and different summary scores derived from raw Rey Auditory Verbal Learning Test (RAVLT) scores (Schmidt, 1996), including RAVLT Immediate [the sum of scores from 5 first trials (Trials 1 to 5)], RAVLT Learning (the score of Trial 5 minus the score of Trial 1), RAVLT Forgetting (the score of Trial 5 minus score of the delayed recall) and RAVLT Percent Forgetting (RAVLT Forgetting divided by the score of Trial 5).

### 2.3 MRI data

The MRI protocol adopted by the ADNI has been previously described (Jack Jr et al., 2008). In summary, the study utilized T1-weighted images acquired from ADNI-approved 3 T scanners, which were cross-sectionally processed using the 2010 Desikan-Killiany atlas with FreeSurfer image analysis suite, version 5.1. The image processing procedures involve several steps, such as motion correction and averaging of multiple volumetric T1 weighted images (Reuter et al., 2010), as well as removal of non-brain tissue using a hybrid watershed/surface deformation procedure (Ségonne et al., 2004). More detailed technical information about these procedures is available in a prior publication (Valerio et al., 2021). The MRI measures included in this study were hippocampus volume, entorhinal cortex volume, middle temporal gyrus volume, fusiform volume, ventricle volume, whole brain volume, and intracranial volume. To account for differences in head size, all MRI measures were normalized by calculating the percentage of these volumes over the intracranial volume. This correction factor allowed for comparisons across participants with varying head sizes, ensuring that the MRI measures were adjusted for individual variations in brain size.

### 2.4 Machine learning models

**Figure 2:**
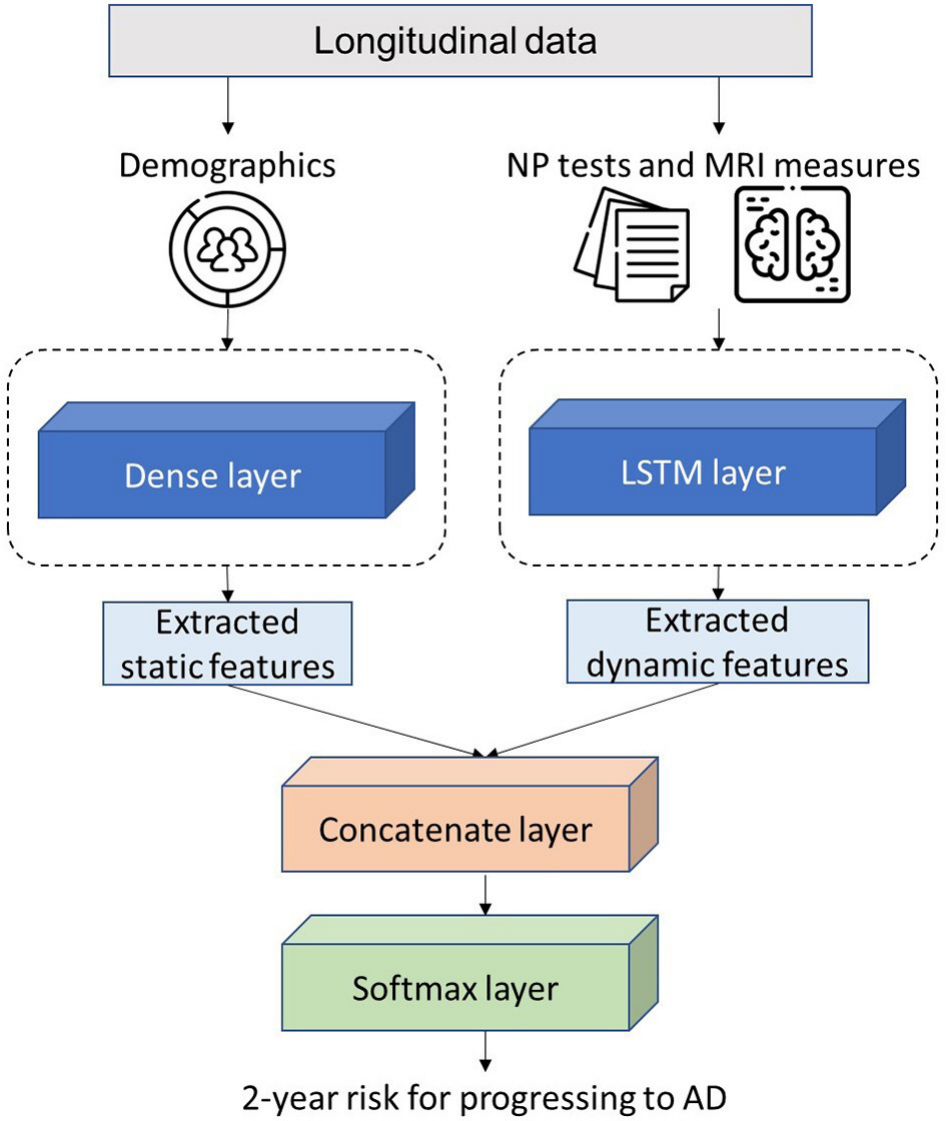
The framework of the LSTM model.

This study developed a LSTM model from multimodal data collected at five time points (baseline, 6, 12, 18, and 24-month) to predict 2-year risk of progression from MCI to AD (Figure 2). LSTM is a type of recurrent neural network that is well-suited to modeling sequential data (Yu et al., 2019). The proposed model consisted of two input layers including an LSTM layer (dynamic layer) and a fully connected dense layer with a rectified linear unit (ReLU) activation function (static layer). Dynamic features (MRI measures and NP tests) collected from five clinical exams were used as the input for the LSTM layer, while the input for the dense layer was static features including demographic information such as age, sex, and education. The output of the LSTM and dense layers were concatenated and passed through a fully connected softmax layer with two output units. We implemented Bayesian optimization (Snoek et al., 2012) to search for the best hyperparameters for the LSTM model. The hyperparameters optimized were learning rate, dropout rate, recurrent dropout rate, number of units in the LSTM layer, and number of units in the dense layer for static variables. The hyperparameter search space was specified with lower and upper bounds for each hyperparameter, including learning rate (0.0001, 0.02), dropout rate (0.05, 0.5), recurrent dropout rate (0.05, 0.5), number of units in the dynamic layer (2, 20), and number of units in the static layer (2, 5). The optimization was performed using two separate 10-fold cross-validation procedures for hyperparameter selection and model performance evaluation. The objective function being optimized was the average area under the receiver operating characteristic (ROC) curve (AUC) across all folds. In each fold, the AdamW optimizer (Loshchilov and Hutter, 2017) was used and the model was trained for a fixed number of epochs with early stopping to prevent overfitting. We further built the model based on the selected best hyperparameters and evaluate its 2-year risk prediction performance.

Random forest, a decision-tree based approach, have gained popularity in the medical field due to their ability to identify complex interactions and nonlinearities of predictor effects (Rigatti, 2017). Given its versatility and performance, we built a random forest model with data collected at the 24-month time point for comparison in this study (Supplementary Figure 1). The model consisted of 100 decision trees. During training, each tree was built using a random subset of the data and considered a subset of features at each node. The predictions of individual trees were then aggregated to generate more accurate and reliable predictions.

### 2.5 Statistical analyses

All continuous variables (i.e., NP test scores and MRI measures) were normalized to have a mean of zero and standard deviation of one. Differences in characteristics between MCI-C and MCI-NC were compared using the Wilcoxon Rank-Sum test for continuous variables and Fisher's exact test for categorical variables. Statistical significance was considered for *P*-values < 0.05. In the analysis, variables with missing values comprising more than 50% were excluded. Multiple imputations using the chained equations (MICE) method (Azur et al., 2011) was used to impute the remaining features with missing values. We performed the DeLong test to examine whether there is a significant difference of performance between the LSTM model and the random forest model.

We constructed two single modality LSTM models using either NP tests or MRI measures to examine the improvement in 2-year progression risk prediction performance with multimodal data. In these models, the structure of the LSTM model was modified by removing the static layer, leaving only the dynamic layer, while keeping the other hyperparameters and optimization methods unchanged.

We further conducted a sensitivity analysis to test the stability of the predictive power of the LSTM model with longitudinal and multimodal data by restricting the analysis to participants who were 65 years or older (*n* = 307, 75 MCI-C) or 70 years or older (*n* = 256, 68 MCI-C) at 24-month.

## 3 Results

### 3.1 Cohort descriptive

This study included 347 ADNI participants (mean age 75 ± 7 years old and 39.8% women) at the index exam (24-month). Their clinical characteristics are presented in Table 1. Participants were followed up for 2 years since the index exam (equivalent to 48-months since the baseline exam), during which 77 (22.2%) were converted to AD. In comparison with those who remained as MCI, patients who progressed to AD were generally older and had lower NP test performance at the index exam. There was no significant difference between the two groups in terms of sex or education. Apart from the ventricle volume, the volumes of all other brain regions were found to be smaller in MCI converters than in MCI non-converters.

**Table 1:** Clinical characteristics of the study participants at index exam (24-month).

**Variable**	**MCI-C (*n* = 77)**	**MCI-NC (*n* = 270)**	***P*-value**
**Demographics**			
Age (years), mean ± SD	76.3 ± 6.5	74.2 ± 7.4	0.034
Women, *n* (%)	33(42.9%)	105(38.9%)	0.620
Years of education, mean ± SD	15.9 ± 2.7	16.0 ± 2.7	0.808
**NP tests, mean** ±**SD**			
FAQ	6.88 ± 5.45	2.01 ± 3.02	<0.001
CDR-SB	2.64 ± 1.17	1.23 ± 0.88	<0.001
ADAS-cog 11	13.45 ± 5.18	7.96 ± 3.71	<0.001
ADAS-cog 13	21.74 ± 7.09	12.90 ± 5.73	<0.001
ADAS-Q4	7.31 ± 2.28	4.31 ± 2.40	<0.001
MMSE	26.21 ± 2.01	28.19 ± 1.91	<0.001
MoCA	21.72 ± 2.85	24.48 ± 3.06	<0.001
LDELTOTAL	3.59 ± 3.95	9.43 ± 4.81	<0.001
mPACC-trailsB	−8.83 ± 3.62	−3.13 ± 3.81	<0.001
mPACC-digit	−9.45 ± 3.97	−3.53 ± 4.48	<0.001
Trails B	121.18 ± 70.40	93.89 ± 42.93	0.002
RAVLT Immediate	28.75 ± 8.31	38.34 ± 11.15	<0.001
RAVLT Learning	2.99 ± 2.55	4.68 ± 2.81	<0.001
RAVLT Forgetting	5.39 ± 2.09	4.51 ± 2.83	0.003
RAVLT Percent Forgetting	82.97 ± 24.92	52.89 ± 33.36	<0.001
**MRI measures, mean** ±**SD**			
Hippocampal volume (%)	0.39 ± 0.07	0.47 ± 0.09	<0.001
Entorhinal cortex volume (%)	0.19 ± 0.04	0.25 ± 0.05	<0.001
Middle temporal gyrus volume (%)	1.17 ± 0.13	1.35 ± 0.16	<0.001
Fusiform volume (%)	1.05 ± 0.18	1.21 ± 0.18	<0.001
Ventricle volume (%)	2.95 ± 1.35	2.49 ± 1.30	0.018
Whole brain volume (%)	64.69 ± 4.55	69.00 ± 6.25	<0.001

### 3.2 Predictive performance of LSTM and random forest models

**Figure 3:**
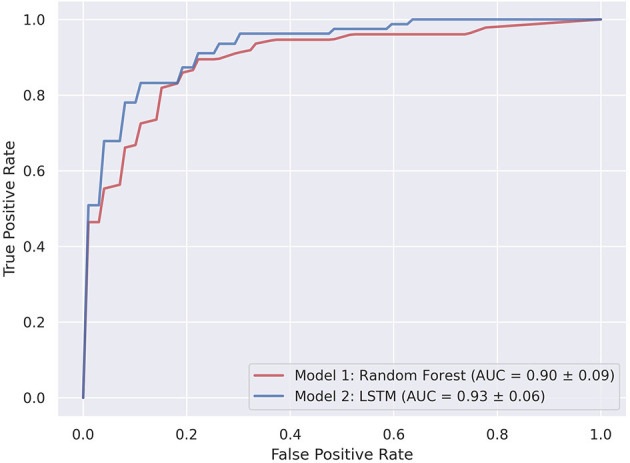
The ROC curves of LSTM and random forest models for predicting 2-year risk of progression from MCI to AD using three data modalities. The blue line represents the LSTM model (baseline, 6, 12, 18, and 24-month), which showed the best performance (AUC 0.93). The red line represents the random forest model with single time point data (24-month) (AUC 0.90).

As shown in Figure 3, the LSTM model, which incorporates longitudinal data (baseline, 6, 12, 18, and 24-month), achieved a significantly higher mean AUC (0.93 ± 0.06) than the random forest model based on the data collected at a single time point (24-month) (0.90 ± 0.09) (DeLong test *P* = 0.039). Additionally, we constructed two random forest models using the data collected across the same five time points (baseline, 6, 12, 18, and 24-month): one using the mean of the data which achieved an AUC of 0.87 ± 0.10, and another using all data which achieved an AUC of 0.89 ± 0.10. This indicates that multimodal data collected at each individual exam provide complementary information for the prediction of MCI-to-AD progression.

**Figure 4:**
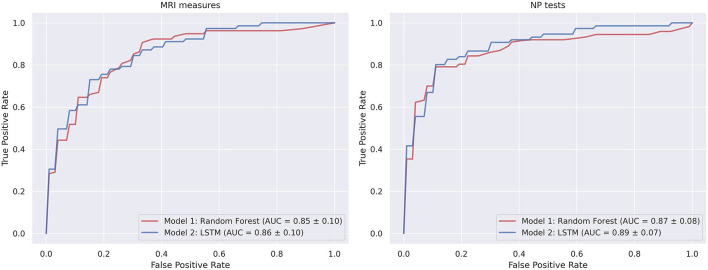
The ROC curves of LSTM and random forest models with single modalities for predicting 2-year risk of progression from MCI to AD.

Figure 4 displays the prediction results of the LSTM and random forest models, using single modalities to predict 2-year risk of progression. The LSTM model utilizing longitudinal data reached the best performance, comparing to an AUC of 0.86 when using MRI measures. The performance of the random forest models using MRI measures had an AUC of 0.85. In terms of NP tests, the LSTM model outperformed the random forest model, achieving an AUC of 0.89 compared to 0.87.

We further performed the sensitivity analysis by restricting study participants across different age groups. The random forest models built on a single time point reached the AUC of 0.89 and 0.87 for participants aged above 65 and 70, respectively. The random forest model using the mean of longitudinal data showed the performance with the AUC of 0.87 and 0.85 for participants aged above 65 and 70, respectively. The LSTM models continued to exhibit the best performance for participants aged above 65 (AUC 0.92) and 70 (AUC 0.90) years old (Supplementary Figure 2).

## 4 Discussion

The progression from MCI to AD is heterogeneous. This study utilized the longitudinal multimodal data from the ADNI and built an LSTM model to predict the 2-year progression risk with an AUC of 0.93. Our results confirmed that incorporating data from multiple time points improved the predictive performance compared to using a single data point across different age-restricted groups.

Timely detection of the progression of MCI to AD is crucial as it enables early interventions such as lifestyle changes, medication, and cognitive training to be implemented, which could potentially delay the onset or slow the progression to AD. As an example, a 2-year delay in the onset of AD could lead to an estimated decrease of approximately 22.8 million cases worldwide by the year 2050 (Brookmeyer et al., 2007; Chuang et al., 2016), underscoring the significant impact even a relatively small delay in disease onset could have on the global burden of AD. Several prior studies have employed cross-sectional data to predict MCI to AD progression using various biomarkers. For instance, MRI measures of hippocampal and entorhinal cortex atrophy have been utilized in the prediction models (Devanand et al., 2007), as well as cerebrospinal fluid biomarkers (Llano et al., 2019). However, as MCI is a heterogeneous syndrome characterized by differences in cognitive profile and clinical progression, predicting the outcome for patients with MCI remains a challenging task.

The life-course perspective emphasizes that health and disease are shaped by a combination of genetic, environmental, and social factors across an individual's lifespan (Kuh and Shlomo, 2014). In recent years, the understanding of AD has shifted toward a gradual accumulation of pathological changes leading to clinical decline, with dementia being the end stage of this process. Studies have suggested that certain factors in midlife, such as LDL cholesterol level (Iwagami et al., 2021), hypertension (Kennelly et al., 2009), and obesity (Pedditizi et al., 2016), are associated with an increased risk of developing AD. Long hierarchical preclinical trajectory of cognitive function decline in dementia were also observed (Verlinden et al., 2016). Multiple MRI measures in midlife were associated with the risk of developing dementia in later life (Debette et al., 2009). These findings are suggestive of a prolonged subclinical phase before the onset of dementia. In this context, the integration of longitudinal clinical exam data from multiple time points allows for a more comprehensive understanding of disease progression. Using past clinical exam data collected from multiple time points is advantageous over relying on a single exam because a single clinical exam may not capture dynamic changes in cognitive and functional abilities.

This study employed LSTM to recognize longitudinal patterns of disease progression that may not be apparent using traditional statistical methods. This feature makes LSTM an appealing method to improve our understanding of disease progression, ultimately leading to better patient outcomes. The model remained stable when restricted to different age groups. In contrast, the random forest models based on a single time point data had relatively large fluctuations in the prediction performance. Our results suggest that accurate dementia prediction becomes more challenging for older populations, especially when using only cross-sectional data. This difficulty arises primarily from the higher prevalence of age-related comorbidities among older individuals, including cognitive decline associated with natural aging. Consequently, distinguishing between early-stage dementia and normal age-related cognitive changes becomes more challenging in this population (Belleville et al., 2017).

The primary clinical indices of neurodegeneration, NP tests and MRI biomarkers each predict risk of progression from MCI to AD, but the combinations of these measures substantially improve prediction performance. Using NP tests for the diagnosis of AD and MCI can be circular, as these conditions are diagnosed based on the severity of cognitive dysfunction. However, the severity of cognitive impairment in MCI can vary widely, and the more severe the impairment, the greater the likelihood of decline to dementia. This is consistent with the findings of our study, which showed that using only NP tests achieved better predictive performance than using only MRI. MRI measures are more likely to be influenced by other factors unrelated to cognitive decline such as atrophy due to aging (Schott et al., 2005). The improved predictive performance from combined use of these measures argues strongly for their inclusion in the clinical investigation of suspected AD.

The present study has several limitations. First, our proposed method relies on longitudinal data and thus requires each subject to have the corresponding modality data across multiple time points. Future research should explore models that can handle uneven exam intervals. Additionally, our study only included longitudinal data collected from five time points within the 2-year period. It would be valuable to further extend the study to include longer follow-up time. Furthermore, our findings are based on a relatively small sample size. Future studies with larger sample sizes should be considered to validate the results. In terms of clinical applicability, the implementation of the model in a clinical setting may present challenges due to its reliance on advanced analytical methods and complex algorithms. Proper interpretation of the model's findings would require specialized knowledge and skills.

In summary, by incorporating longitudinal multimodal data, our LSTM model achieved superior performance in predicting progression from MCI to AD compared to using data collected at a single time point. This demonstrates that a longitudinal and comprehensive assessment of cognitive and functional decline can serve as a valuable data resource in improving the predictive capability of disease progression. Future utilization of life course information will further improve the prediction capability for MCI to AD progression.

## Data availability statement

Publicly available datasets were analyzed in this study. This data can be found here: https://adni.loni.usc.edu/data-samples/access-data.

## Ethics statement

The studies involving humans were approved by Alzheimer's Disease Neuroimaging Initiative. The studies were conducted in accordance with the local legislation and institutional requirements. Written informed consent for participation was not required from the participants or the participants' legal guardians/next of kin in accordance with the national legislation and institutional requirements.

## Author contributions

HD: Writing – original draft, Writing – review & editing, Conceptualization, Methodology, Data curation, Formal analysis. BW: Writing – review & editing. AH: Writing – review & editing. MM: Writing – review & editing. TA: Writing – review & editing. RA: Writing – review & editing. HL: Writing – review & editing, Conceptualization, Methodology.

## Footnotes

^1^ https://adni.loni.usc.edu/
