# Supplementary material for: Prediction of progression from mild cognitive impairment to Alzheimer's disease with longitudinal and multimodal data
Source: Front Dement. 2023 Nov 24;2:1271680. doi: 10.3389/frdem.2023.1271680 (PMC11185839; doi:10.3389/frdem.2023.1271680)

Supplementary Material

Prediction of progression from mild cognitive impairment to Alzheimer’s disease with longitudinal and multimodal data

Huitong Ding, Biqi Wang, Alexander P Hamel, Mark Melkonyan, Ting F. A. Ang, for the Alzheimer's Disease Neuroimaging Initiative, Rhoda Au, Honghuang Lin*

*** Correspondence:** honghuang.lin@umassmed.edu

# Supplementary Figures

**Supplemental Figure 1.** The architecture of random forest model with cross-sectional data collected at the 24-month examination.

**
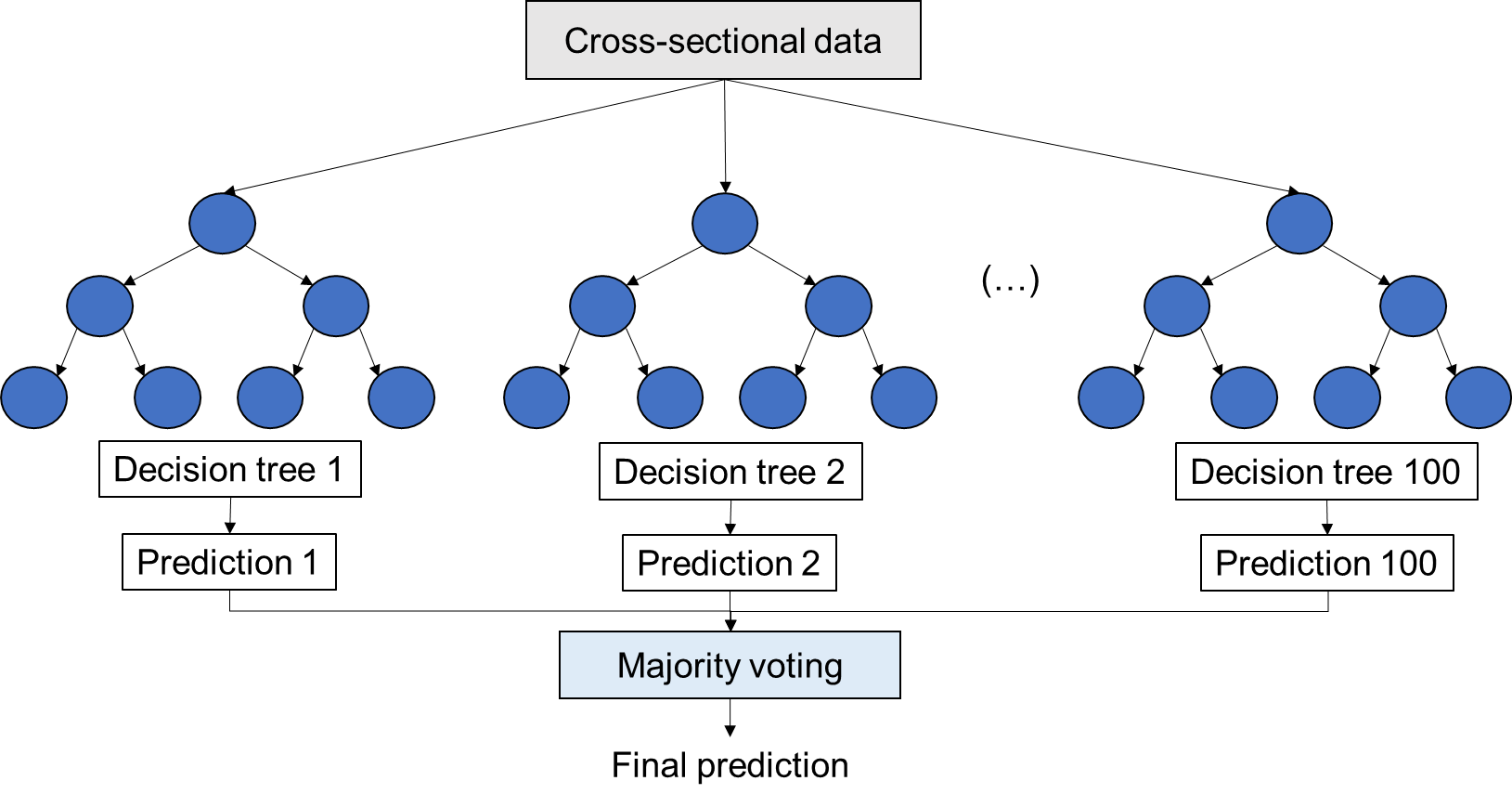
**

**Supplemental Figure 2.** The ROC curves of LSTM and random forest models for predicting 2-year risk of progression from MCI to AD for participants aged 65 years older, and participants aged 70 years older.


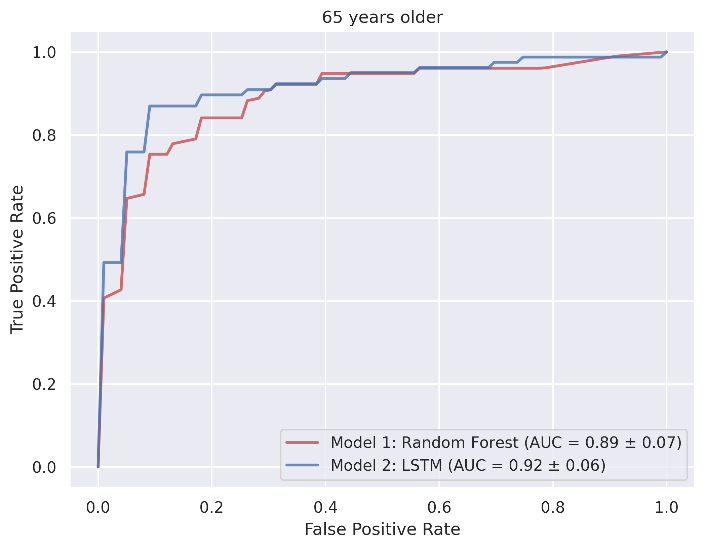

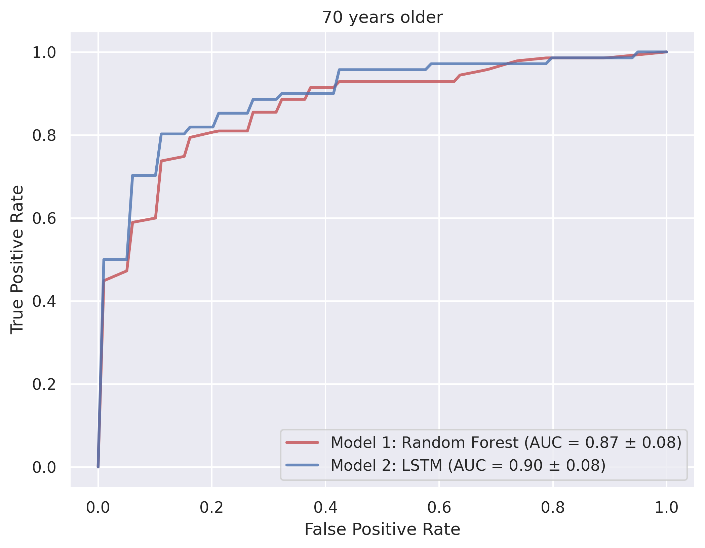

Supplement: Supplementary file 1 [file Data_Sheet_1.docx]
